# Developmental processes in ageing: the role of growth regulation

**DOI:** 10.1098/rstb.2025.0329

**Published:** 2026-11-12

**Authors:** Idálio de Jesus Viegas, João Pedro de Magalhães

**Affiliations:** Genomics of Ageing and Rejuvenation Lab, Department of Inflammation and Ageing, College of Medicine and Health, University of Birmingham, Birmingham, B15 2WB, UK

**Keywords:** development, growth restriction, antagonistic pleiotropy, geroscience, longevity

## Abstract

The underlying causes of ageing remain elusive, as evidenced by the numerous coexisting theories attempting to explain its origin and progression. Developing a robust theoretical framework will not only improve our understanding of this fundamental biological process but also guide the design of interventions targeting the ageing process. Here, we revisit the hypothesis originally proposed by Bidder that the growth-restriction processes—responsible for suppressing growth during development and establishing adult body size—remain active throughout adulthood, potentially acting as a primary driver of ageing. We explore how persistent growth suppression can induce ageing phenotypes, such as organ atrophy and degeneration. Furthermore, we examine supporting observations from comparative studies and experiments in model organisms and address the current limitations of this model, with the expectation that this framework will be further developed and assessed in future research.

This article is part of the theme issue ‘Developmental circuits in ageing’.

## 1. Introduction

Understanding the drivers of ageing has long been a goal in biology, with renewed urgency given population ageing and the rising burden of age-related disease. Mechanistic theories of ageing are often divided into damage-based models and programme-based models, with the former dominating contemporary research [1,2]. Yet primary drivers of human ageing remain elusive, and why we age remains a fundamental biological question.

Programme-based theories can be further subdivided into those proposing that ageing evolved for an adaptive purpose [3,4] and those suggesting that ageing results from evolutionary neglect, driven by gene regulatory programmes or the continued action of developmental processes beyond their optimal window [5]. Interest in programmatic processes has resurged with findings that caloric restriction (CR), mutations affecting the growth hormone (GH) pathway and rapamycin (Rapa) extend lifespan in mice [5]. Furthermore, the development of epigenetic clocks has intensified interest in developmental processes as possible drivers of ageing, as clock predictions cover the entire lifespan [6,7].

If ageing results from the continued action of developmental programmes, it is necessary to identify the specific developmental processes and pathways that drive ageing phenotypes. One hypothesis is that ageing is associated with or even driven by growth restriction and suppression. The hypothesis that growth regulation promotes ageing was first proposed around a century ago by Bidder [8]. In that work, Bidder suggested: ‘*senescence is the after-result of the mechanism which secures specific size … This mechanism may be called the regulator, avoiding the word “inhibitor” so as not to connote a physiological assumption. However ignorant we are of its nature, its action is traced in anthropometric statistics: a steady diminution in growth rate from a maximum at puberty to vanishing-point in the twenties*… *I have suggested that senescence is the result of the continued action of the regulator after growth is stopped*’ [8, p. 583]. Bidder conceptualized that the process responsible for the gradual deceleration of growth towards adult size persists throughout adulthood, thereby driving the onset of senescence. While the mechanisms regulating growth were largely unknown in Bidder's time, most of the processes governing tissue expansion and final body size remain poorly understood today, despite significant advances across multiple biological levels. Bidder termed this regulatory deceleration ‘negative growth’, suggesting it represented a seamless continuation of the normal developmental programme into adulthood: ‘*This negative growth is not the manifestation of a weakness inherent in protoplasm or characteristic of nucleated cells; it is the unimportant by-product of a regulating mechanism necessary to the survival of any race of swiftly moving land animals, a mechanism evolved by selection and survival as have been evolved the jointing of mammalian limbs, and with similar perfection*’ [8, p. 585].

In the decades following Bidder's proposal, other researchers advanced similar concepts. For instance, influenced by the discovery of the Hayflick limit, Martin [9, p. B172] proposed that: ‘*While the process of clonal attenuation may be adaptive during early life, it almost certainly has nonadaptive, deleterious consequences postreproductively, when the force of natural selection declines. Thus, it may be responsible for various tissue atrophies.’* Additionally, based on studies of the mechanisms governing tissue growth and the hypothesis that conserved processes drive a coordinated decline in proliferation across multiple organ systems, Lui [10, p. 641] proposed: ‘*that this genetic program continues to progress into later adult life, eventually suppressing proliferation to levels below those needed for tissue renewal, thus contributing to aging*’.

Here, we revisit the hypothesis that ageing is a result of the continuation of growth restriction, integrating recent findings to frame it within modern geroscience.

## 2. How can growth restriction promote ageing?

Growth-restricting signals are responsible for tempering growth rate during development, leading to a progressive deceleration until adult tissue size is reached. According to the Bidder hypothesis, this suppressive signalling does not terminate upon reaching maturity but remains active throughout adulthood. These growth-restriction processes are linked to biological turnover—the nearly universal process of continuous synthesis and degradation. Turnover occurs across multiple levels of biological organization; even in the absence of damage, cellular components like RNAs and proteins are constantly renewed [11]. In many tissues, this process extends to entire cells and extracellular matrix components, such as bone. While turnover rates vary significantly across molecules and cell types, the genome remains a rare exception, as it is replicated during division rather than undergoing continuous degradation [11]. Maintaining a constant tissue size thus requires an equilibrium between production and degradation. Growth occurs when production exceeds degradation, whereas tissue loss (or negative growth) results from the inverse. Consequently, growth-restricting signals that lower growth rates are expected to function by limiting cell proliferation and reducing the synthesis of new biomolecules.

During early embryonic stages, characterized by high growth rates, most cells are actively proliferating. As development progresses through the fetal stage, growth rates peak and then begin to decline [12]. Postnatally, this deceleration continues, with tissue growth slowing progressively until net growth ceases and definitive adult size is established [13]. This downward trajectory suggests that growth-restrictive signalling is not only activated during development but also progressively strengthened, reducing proliferative and biosynthetic activity. However, if the growth-rate trajectory continues its downward trend and falls below zero, the system enters a state of ‘negative growth’, as envisioned by Bidder and consistent with antagonistic pleiotropy [14]. In this state, the rate of biomass degradation outpaces production, leading to a net loss of tissue mass. This transition into negative growth provides a mechanistic explanation for the progressive tissue atrophy observed during ageing.

The growth rate trajectory can be visualized through a lifelong growth-rate curve (figure 1), which maps the rate of biomass production from development to old age and can be categorized into three phases. In phase 1, the growth rate is positive and accelerating; tissue expansion occurs as biomass production exceeds degradation. In phase 2, following the developmental peak, the growth rate remains positive but undergoes progressive deceleration. Tissues continue to expand at a diminishing rate until reaching the renewal threshold, where production and degradation achieve a homeostatic equilibrium and net growth ceases. This growth rate decline during phase 2 suggests that an endogenous process must actively suppress tissue growth. In phase 3, the growth rate enters a negative trajectory where degradation outpaces production. This imbalance results in a net loss of cellular, intracellular and extracellular components, manifesting as the tissue atrophy characteristic of ageing. Theoretically, phase 3 results from sustained, high-level growth-restriction signalling—a concept that aligns with the ‘regulator’ hypothesized by Bidder as a primary driver of ageing. Additionally, the growth-suppressive signals could lock the tissue in a state that prevents growth, even in the absence of the signalling that halted growth in the first place. Crucially, this curve applies not only to the whole organism but also to the specific trajectories of individual tissues, even though these tissues may vary in their peak growth timing or rates of deceleration. While phases 1 and 2 can be directly assessed through physical measurements of size, phase 3 represents a theoretical extension of this trajectory into adulthood.

**Figure 1.**
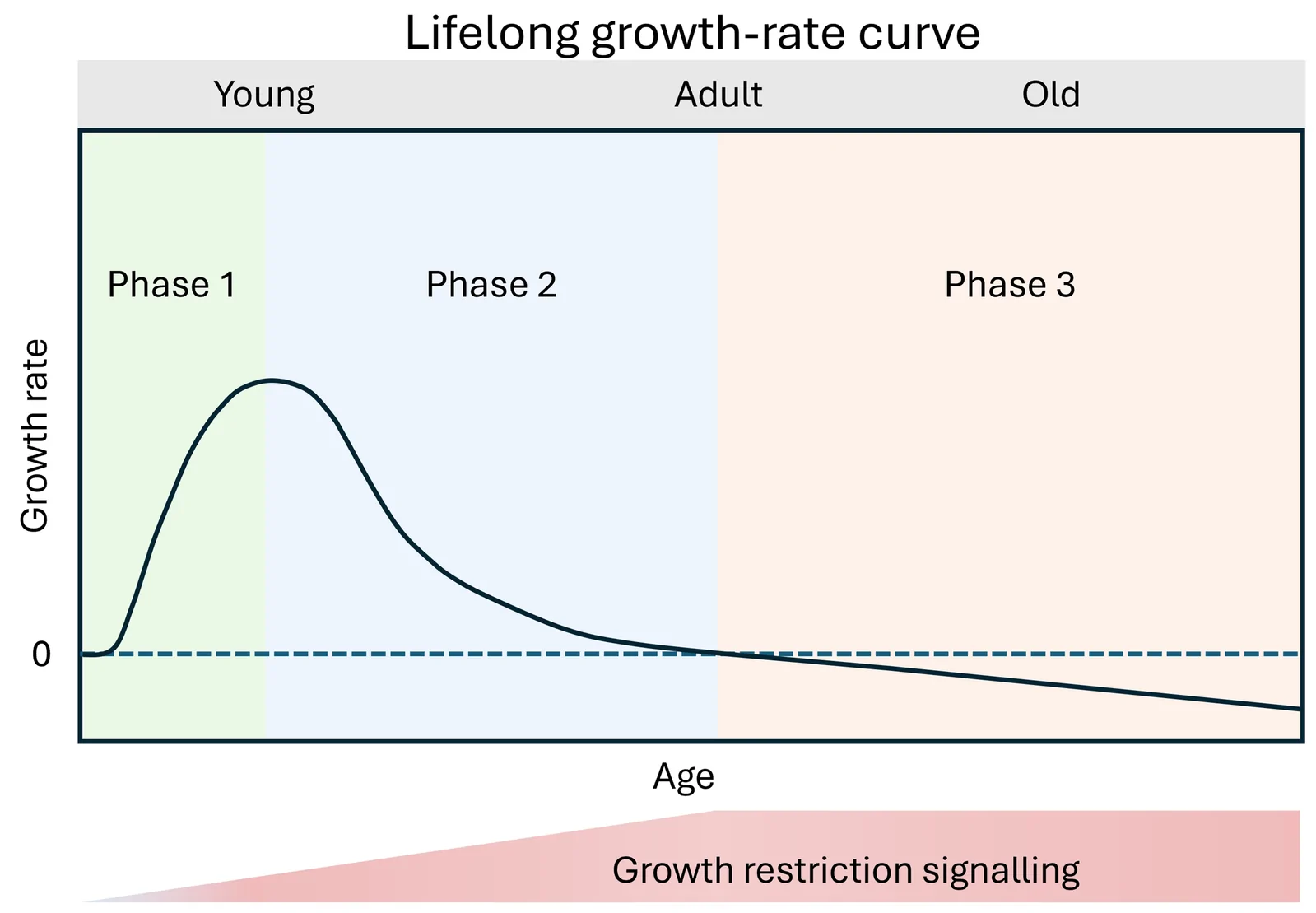
Lifelong growth-rate curve. Schematic representation of a generalized tissue growth-rate trajectory across the lifespan. The *y*-axis represents growth rate (rate of tissue mass change over time) and the *x*-axis represents age. The lower panel illustrates the progressive increase in growth-restriction signalling intensity, leading to a decline in growth rate that persists at high levels throughout adulthood. The curve is divided into three phases: Phase 1, growth rate is positive and increasing (accelerating tissue expansion); Phase 2, growth rate is positive but decreasing (growth deceleration); and Phase 3, negative growth rate, indicating tissue atrophy. Phase 3 represents a theoretical continuation of the trajectory established in earlier stages. Although theoretical, this model may approximate the general growth dynamics of multiple tissues, with tissue-specific differences in kinetics, steepness and timing.

Based on the growth-restriction model, ageing results from insufficient cell proliferation and/or molecular biosynthesis to maintain homeostatic turnover. As a result, tissue function gradually becomes compromised. Because tissue performance depends on both the number and the functionality of its constituent cells, the combination of reduced cell density and impaired molecular renewal leads to systemic decline. We can visualize these tissue shifts across distinct life stages (figure 2). In young tissues, highly active stem and progenitor cells generate an increasing number of functional, differentiated cells to support growth. As the growth rate slows, the frequency of proliferating cells and biosynthetic activity declines. By adulthood (peak functionality), an equilibrium exists where stem/progenitor activity balances cell loss, maintaining a stable population of functional cells. The persistent decline in proliferation and biosynthesis eventually results in aged tissues characterized by reduced cellularity. The remaining cells exhibit a gradual loss of function, driven largely by diminished biosynthetic activity. Owing to the interdependence of biological systems, the functional decline caused by growth restriction could be amplified by self-reinforcing ‘vicious cycles’, as proposed for other ageing phenotypes [15].

**Figure 2.**
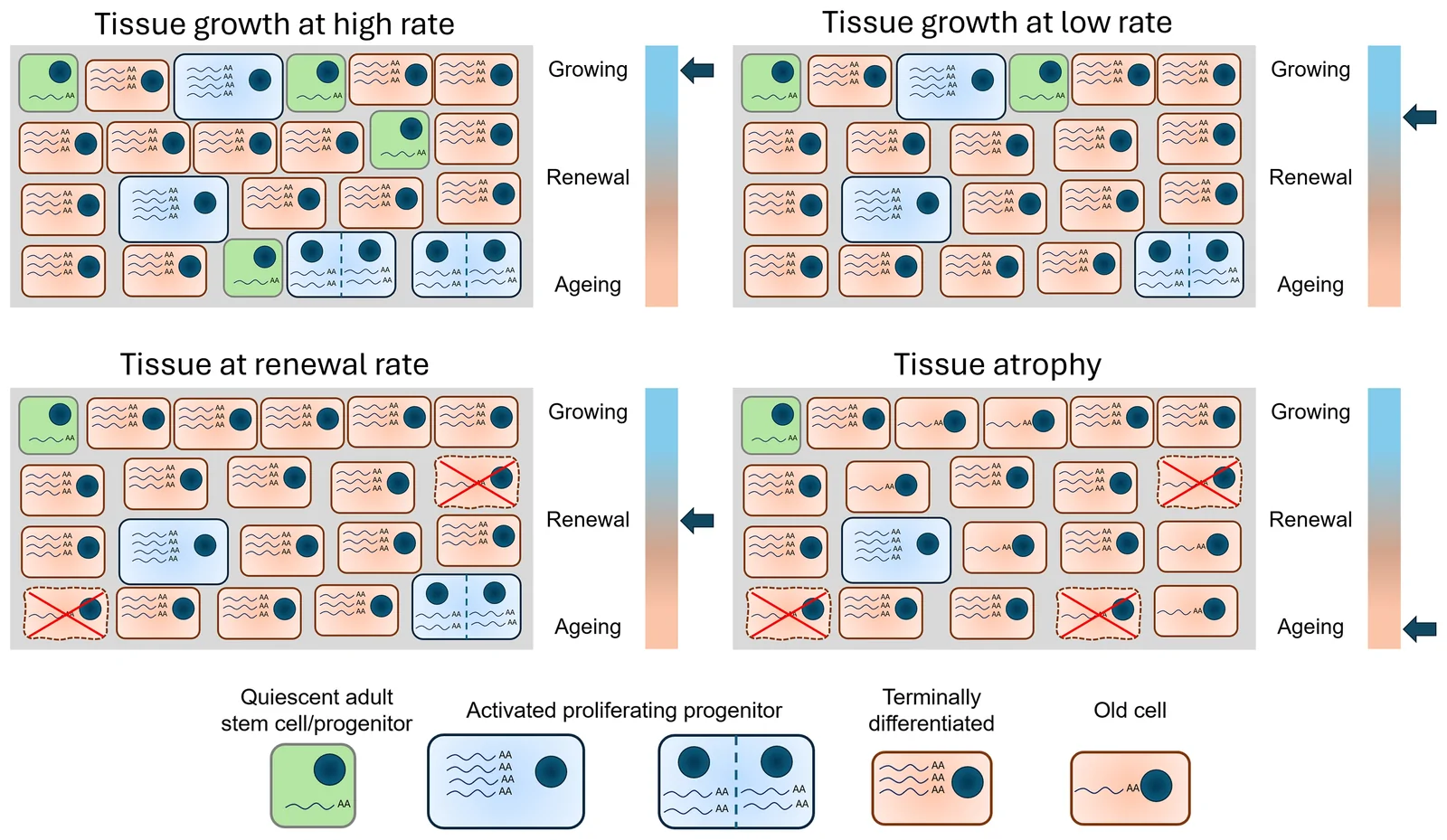
Tissue changes with age according to the growth-restriction model. Schematic representation of tissue dynamics across the lifespan. In this simplified model, tissues are composed of stem/progenitor cells (SC) and differentiated functional cells. Activated SC increase biosynthesis (cellular growth) and divide, generating new cells to support tissue expansion and homeostatic turnover. During early life (top left), tissue growth is rapid, with many SC actively growing and dividing. As development progresses (top right), tissue growth continues but at a reduced rate, with fewer progenitor cells actively proliferating. At young adulthood (bottom left), tissue growth reaches net equilibrium (renewal stage): progenitor cell proliferation balances normal cellular turnover, resulting in stable tissue mass and maximal functional capacity, dominated by differentiated functional cells. With further ageing (bottom right), continued growth restriction leads to reduced progenitor cell growth and proliferation, insufficient to fully compensate for cell loss owing to normal turnover or other mechanisms, such as apoptosis, necrosis and immune clearance and leads to reduced biosynthesis in differentiated cells, resulting in diminished tissue function and progressive atrophy. This simplified model illustrates how sustained growth restriction over time can drive age-associated tissue decline.

Overall, we proposed a process by which the trajectory of growth restriction—extending from development into adulthood—serves as a primary causal factor for the emergence of ageing phenotypes. In this view, the Bidder hypothesis can be updated as: ageing/senescence results from a continuous growth-restriction process that pulls the growth-rate trajectory below the threshold required for physiological renewal, leading to ‘negative growth’.

## 3. Growth regulation: how is growth regulated?

How is growth regulated and how is adult body size determined? How do distinct tissues sense when they have reached their target dimensions and subsequently cease expansion? Furthermore, how is growth coordinated across distant organ systems to ensure symmetrical and proportional development, such as the identical lengths in bilateral limbs? On an evolutionary scale, why are some species small while others achieve enormous mass, spanning orders of magnitude? Despite being fundamental to our understanding of biology, growth regulatory processes remain poorly understood.

A component of growth regulation is the growth hormone (GH/IGF1) axis. While elevated GH activity correlates with larger body size and reduced signalling results in smaller stature, GH explains only a fraction of adult size determination. Evidence suggests that growth-restrictive processes override GH stimulation, as growth rates decline during juvenile development even when GH levels remain high [13]. Body size is a function of both cell number and cell volume; consequently, growth regulation necessitates the precise control of cell growth, proliferation, survival and apoptosis. Deciphering the coordination and regulation of these processes requires understanding the integration of local and systemic cues, including the interplay between stimulatory and inhibitory signals [13,16,17]. Furthermore, cellular lineage and identity influence proliferative capacity; proliferation is usually inversely coupled to differentiation, where terminal differentiation typically triggers the cessation of the cell cycle [18].

Integrating various lines of evidence regarding body size regulation, Lui & Baron [13, p. 429] proposed a negative feedback model in which tissue expansion inherently triggers its own limitation: ‘*growth causes the down-regulation of a large set of growth-promoting genes, which in turn causes body growth to progressively slow and eventually cease, thus setting a fundamental limit on adult body size*’. This model is characterized by an elegant architecture analogous to classic homeostatic feedback loops. However, the precise regulatory mechanisms driving this loop remain largely uncharacterized. Several potential sensing mechanisms—including the monitoring of organ mass, functional output and cumulative cell division count—were discussed and proposed to explain how the system determines when target size is reached [13]. Overall, this negative feedback framework provides a robust conceptual foundation for investigating the regulation of body size.

A compelling framework for understanding growth regulation and its relationship to ageing is the theory of organisms [19]. This theory is founded on three core principles: the default state of proliferation (characterized by variation and motility), the principle of variation and the principle of organization. It posits that the most fundamental and evolutionarily ancient state of a cell is one of continuous proliferation. In the context of multicellularity, this ‘default’ drive must be actively constrained to ensure precise tissue organization, functional integrity and size control [19]. By integrating cell biology with evolutionary theory, this framework highlights that the suppression of proliferation is a prerequisite for multicellular development. Furthermore, it offers a novel perspective on oncogenesis, suggesting that cancer arises when these organizational constraints are disrupted [20].

It is noteworthy that the theory of organisms emphasizes the necessity of suppressive growth control to constrain the default proliferative state of cells—a concept congruent with growth restriction models governed by negative feedback loops. This perspective aligns with evolutionary transitions: while early life was unicellular, the emergence of multicellularity would have required the imposition of functional constraints and the active coordination of cell proliferation. Furthermore, indeterminate growth—where organisms continue to grow throughout their lifespan—has been proposed as the ancestral growth pattern in animals [21]. Additionally, it has been suggested that the phenomena of negative or negligible senescence may result from such indeterminate growth trajectories [22].

The regulation of tissue growth—governed by negative feedback loops and constraints on the default proliferative state— should be based on a complex architecture of sensing and signalling. While these interactions are still being decoded, some pathways have emerged as mediators, including the Hippo pathway, the Wnt/beta-catenin and TGF-β [23–25] and recently, bioelectric signals [26]. These signalling cascades drive shifts in cellular state that could be stabilized by epigenetic remodelling, such as DNA methylation and histone modifications [13,27]. These pathways likely operate as an integrated network, with their relative contributions varying by context [28–30]. Additionally, growth is a balance between inhibitory signals and positive stimulatory factors, such as FGF, VEGF, EGF and TGF-β [31]. Additionally, comparative genomics analyses of species with different body sizes could provide a strategy for identifying potential regulators of body size. Using this approach, comparative genomics in Carnivora found around 300 genes associated with body size, which were functionally enriched for metabolism, obesity, development and tumour suppression [32]. Genomic analysis of cetacean evolution (a group with species that reach huge body sizes) suggests an association of large body size with genes related to tumour suppression, immunity, growth and metabolism [33–36]. Genomic analysis in Serpentes (a group with large body size variation) identified metabolic and developmental genes associated with body size [37]. Evaluation of the genome of the capybara, the largest rodent in relation to other rodents, suggested that genes potentially associated with its larger body size include functions such as postnatal bone growth regulation and musculoskeletal development [38]. These comparative studies start to reveal the underlying pathways that could explain the regulatory processes determining body size in animals.

Overall, the regulation of growth in animals remains poorly understood, and several models are still being developed to explain this process. Current frameworks—including those based on negative feedback from growth itself and those that view cell proliferation as a default state—emphasize the importance of inhibitory mechanisms and constraints as major determinants of tissue growth regulation. A deeper understanding of growth regulation will be essential to better assess how growth restriction influences the ageing process.

## 4. Evidence of a growth restriction effect on ageing

What evidence supports Bidder's hypothesis that growth-restriction mechanisms causally contribute to ageing? A primary observation is the correlation between body size and maximum lifespan across species, where larger animals generally exhibit greater lifespan [39]. This pattern persists across mammals, birds and reptiles, with notable exceptions—such as bats—that live longer than their size predicts. Critically, larger species typically undergo a slower development; growth rates and developmental timing are negatively correlated with lifespan [39], including in plants [40]. While these correlations do not prove a direct physiological link, they suggest that shared regulatory mechanisms may govern both growth and ageing. Three lines of evidence further reinforce this connection: the prevalence of tissue atrophy during ageing, the systemic reduction in biomolecular biosynthesis during ageing, and the effects observed when growth-associated genes are experimentally manipulated.

### (a) Ageing is associated with tissue atrophy

While growth is characterized by tissue production, atrophy is the opposite: a reduction in tissue mass and cellularity. Therefore, if growth-restriction signalling remains active beyond the attainment of adult size, it is expected to eventually drive tissue atrophy. Consistent with this, data reveal atrophy across multiple organ systems during human ageing (table 1). At the organismal level, height gradually decreases with age and individuals with lean body compositions typically maintain or further lose lean mass over time [99]. Notably, total body mass and BMI (body mass index) are often unreliable indicators of atrophy, as age-related increases in adiposity and fluid retention—particularly in modern sedentary populations—can mask the underlying loss of functional tissue. Furthermore, BMI is influenced by varying levels of physical activity [99]. Therefore, examination of individual tissues and organs provides a more accurate assessment of age-associated atrophy.

**Table 1.** Evidence of tissue atrophy with ageing.

organ / tissue	description
thymus	Undergoes thymic involution, characterized by a reduction in thymic cells—particularly thymic epithelial cells—and replacement with adipose tissue. This process begins early in life (around 1 year in humans) [41].
brain	Total brain mass and volume decrease with age, including grey matter, white matter and cortical thickness. This decline is partly owing to reduced numbers of cells and cellular shrinkage, including neuronal shrinkage and reduced dendritic complexity [42–46].
skeletal muscle	Muscle mass declines with age owing to loss of muscle fibres and a reduction in fibre size, a condition known as sarcopenia. Evidence suggests that reduced fibre size is the dominant contributor to muscle mass loss [47–50].
bone marrow	Ageing is associated with a decline in red (hematopoietic) marrow and an increase in fatty marrow. Despite reduced cellularity, the number of hematopoietic stem cells appears to increase with age [51].
bone	Bone density decreases with age, increasing fragility and fracture risk [52].
kidney	Total kidney volume declines with age, primarily owing to reduced cortical volume. Medullary volume increases in men and initially in women, but declines in women around 50 years of age. Nephron number, the functional unit of the kidney, decreases with age [53–56].
skin	Both dermal and epidermal thickness decline with age. Aged skin shows reduced numbers of melanocytes, Langerhans cells, endothelial cells and pericytes, along with decreased extracellular collagen density. Imaging studies also reveal architectural atrophy, including reduced stromal cell density, shrinking hair and sebaceous glands, shortened dermal white adipose tissue and reduced smooth muscle length [57–59].
pancreas	Total and parenchymal pancreas volume decrease with age, while pancreatic fat content increases. Pancreatic beta cell proliferation decreases [60,61].
gastrointestinal tract	The number and volume of interstitial cells of Cajal decrease with age. In the colon, goblet cell numbers per crypt may decline with age, although this is less evident when normalized by mucosal area. Intestinal (duodenum) villus length decrease with age [62–64].
liver	Maintains regenerative capacity and high cellular turnover throughout life. Nevertheless, liver size declines with age, hepatocytes show increased polyploidy and functional changes occur, though the liver remains relatively resilient to ageing [65–68].
cartilage & intervertebral disc	Knee cartilage thickness declines with age in most regions, and tibial cartilage volume decreases. Intervertebral disc thickness also declines with age [69–72].
adipose tissue	Evaluation of fat atrophy is complex owing to lifestyle effects. With age, visceral fat increases, while subcutaneous fat and brown adipose tissue tend to decrease [73–76].
ovary	The number of non-growing follicles (ovarian reserve), established before birth, declines continuously with age. Ovarian size increases early in life, peaks, then declines from around 20 years of age onward [77,78].
testes	Testicular size increases until the 30s, remains stable through midlife and declines at older ages (from the 60s) [79].
blood	Red and white blood cell (WBC) concentrations generally decline with age. WBC subtypes show sex-specific trajectories: lymphocytes decrease overall, with a transient midlife increase in females, monocytes and neutrophils increase with age, with a temporary midlife decrease in females. Circulating WBC counts are highly sensitive to environmental and inflammatory states [80,81].
heart	Left ventricular wall thickness increases with age, though total left ventricular mass may remain stable. Some evidence suggests cardiomyocyte loss in males. The sinoatrial node shows signs of atrophy. Associations between skeletal muscle loss and reduced cardiac size have led to the concept of ‘cardio-sarcopenia’. Overall, there is limited evidence for generalized cardiac atrophy, suggesting changes may be localized [82–89].
lung	Evidence indicates a reduction in the number of small airways with age. Transcriptomic deconvolution suggests decreased alveolar epithelial cells and increased fibroblasts and endothelial cells [90,91].
eye	Central corneal thickness and corneal endothelial cell density decrease with age, accompanied by increased cell size. Retinal ganglion cell numbers decline, along with retinal thinning and reduced capillary density, although retinal weight and protein content may be preserved [92–94]. Photoreceptor density in the retina decreases with age [95].
ear (cochlea)	Outer hair cells decline with age, with an even greater reduction in associated neural cells [96].
prostate	Unlike most tissues, prostate mass and size tend to increase with age [97,98].

Analyses of histological samples from the GTEx database identified atrophy and microvascular rarefaction as features that increase with age across multiple organs. By contrast, features associated with epithelium and hyperplasia showed a decreasing trend with age in many organs [100]. Additionally, comparative studies of organ mass and cellular turnover suggested that many human tissues suffer a significant loss of functional mass with age—a phenomenon most pronounced in high-turnover tissues [101]. Another study reported age-related atrophy in several human tissues alongside regions of excessive cell growth (hyperplasia) and proposed that hyperplasia may represent a compensatory response to tissue atrophy [102]. Measuring cell proliferation *in vivo* is challenging and commonly used markers, such as Ki67, have limitations [103]. They can be used as proxies for identifying proliferating cells. Using such proliferation markers, an age-related decline in proliferating cells has been reported in several high-turnover human tissues (in the epithelium), such as the colon, oesophagus, duodenum and posterior ethmoid [104]. Altogether, these observations show that atrophy is a common feature of ageing across multiple tissues.

### (b) Protein and mRNA synthesis with age

Tissue growth depends on biosynthetic rates exceeding those of degradation; therefore, signalling that restricts growth is expected to lower cellular biosynthetic activity. Because proteins and RNAs are major cellular components, their abundance not only reflects cell function but can also serve as a proxy for overall biosynthetic activity during ageing. Whole-body measurements in humans indicate that translation rates decline from development to old age [105]. Consistent with this, reduced rRNA gene activity has been documented in ageing human skin fibroblasts [106]. Additionally, measurements of mitochondrial translation in human muscle have been shown to decrease with age [107]. A gene expression meta-analysis further suggested a general decline in transcriptional activity with age [108]. Furthermore, a systematic analysis of common transcriptomic ageing signatures identified a consistent downregulation of protein synthesis machinery [109]. Together, these findings suggest that both mRNA and protein synthesis decrease with age, suggesting a systemic transition towards reduced biosynthetic activity.

### (c) Manipulations of growth-associated genes

Further evidence for the relation between growth restriction and ageing is found in interventions that locally stimulate tissue growth. Manipulation of growth factors or regulators of proliferative pathways has been shown to rescue or mitigate ageing phenotypes. For example, fibroblast growth factor 21 (FGF21) has been demonstrated to counteract age-related thymus involution in murine models [110,111]. Similarly, increasing systemic levels of vascular endothelial growth factor (VEGF) mitigates microvascular rarefaction and extends lifespan in mice [112]. Furthermore, VEGF-A has been shown to restore cell proliferation rates in ageing human skin samples [113] and enhance muscle regeneration capacity in aged mice [114].

Beyond growth factors, the regulation of cell proliferation provides further evidence linking growth restriction to ageing. Systemic inactivation in adult mice of the Myc transcription factor, a regulator of macromolecular biosynthesis and cellular proliferation [115], triggers the premature emergence of ageing phenotypes. Interestingly, while these mice appear ‘older’ phenotypically, they exhibit extended lifespans owing to a significant reduction in cancer incidence—a major cause of mortality in this strain [116]. Similarly, FoxM1, a transcription factor that promotes the expression of cell-cycle genes, declines with age [117]. Experimental *in vivo* restoration of FoxM1 in mice has been shown to recover lost cellular functions, delay the onset of age-associated pathologies and extend healthspan [118]. Collectively, these findings suggest that the functional decline of ageing tissues is, at least in part, a reversible consequence of suppressed growth signalling.

The evidence linking growth restriction to ageing is drawn from diverse observations and experiments. These include the relationship between body size, growth rate and lifespan across species; the observation of tissue atrophy across multiple tissues; the age-related decline in global biosynthetic output and the impact of manipulating growth pathways on ageing phenotypes. While these phenomena could arise from growth restriction-independent biological processes, their collective alignment suggests a parsimonious explanation: that the same processes responsible for restricting growth during development continue to drive the phenotypic decline of ageing. Consequently, this framework offers a plausible direction for future research into the fundamental causes of ageing.

## 5. Growth restriction model in the context of current geroscience

### (a) Relation with other models

The hypothesis presented here that growth restriction serves as a causal driver of ageing provides an explanation for ageing phenotypes, such as tissue atrophy and diminished biosynthesis. While this framework does not exclude other processes, such as models based on stochastic accumulation of molecular damage, we consider it plausible, as the growth-restriction model requires only the assumption that the developmental trajectory of declining growth persists into adulthood. By contrast, damage-based models typically assume that stochastic damage progressively accumulates, reaching levels in adulthood that interfere with the normal physiological function of cells. Additionally, ageing can be multifactorial; therefore, both processes could operate in parallel. In this view, parallel theories of ageing may account for phenotypes that are difficult to explain through growth restriction alone, such as the age-related increase in prostate size [97]. We propose that while growth restriction is a plausible driver of ageing, it is not the sole cause; rather, it is one of various potential contributing causes that lead to specific, but not all, observed ageing phenotypes.

While growth restriction may explain tissue atrophy and declining biosynthesis, other theories offer alternative causes for these same processes. A prominent example is tumour suppression: because cancer is driven by aberrant cell proliferation, the mechanisms that evolved to suppress tumourigenesis may limit proliferation in healthy tissues, thereby contributing to age-related atrophy [119,120]. Similarly, tissue constraints on excessive cellular activity may inadvertently drive ageing by progressively depleting cell function or numbers over time. This concept, described as ‘selective destruction’, suggests that the very protective barriers meant to avoid overactivity disorders eventually impair tissue function [121,122].

The relationship between the growth restriction hypothesis and the hyperfunction hypothesis is noteworthy. On the one hand, the two concepts appear distinct and opposing. Blagosklonny's hyperfunction hypothesis proposes that ageing is driven primarily by mTOR hyperfunction—where excessive cellular growth continues after organismal growth has ceased [123,124]. Conversely, the growth restriction hypothesis suggests that ageing stems from insufficient tissue and cellular growth. One possible interpretation is that both processes operate in parallel, with each driving different ageing phenotypes. For instance, generalized atrophy is more readily explained by growth restriction. On the other hand, through the lens of a broader hyperfunction theory (any excessive biological function), growth restriction itself can be viewed as a case of hyperfunction—specifically, the exaggerated activity of growth-inhibitory signalling. Ultimately, how these two hypotheses relate depends on one's perspective: whether hyperfunction is defined narrowly around mTOR-driven cellular growth or more broadly as any overactive biological process.

### (b) Limitations of the growth restriction model

The growth restriction model proposes that the mechanisms limiting tissue growth at the end of development eventually contribute to ageing phenotypes, such as atrophy. However, certain observations challenge this model. Two primary lines of evidence appear contradictory: interventions that reduce growth yet extend lifespan and long-lived small animals, such as bats.

### (c) Reconciling the growth restriction model with lifespan-extending interventions and small, long-lived animals

#### (i) Growth and lifespan differences between individuals and species

Common interventions that reduce growth while extending lifespan, such as CR [125], Rapa treatment [126] and mutations in GH signalling (GH/IGF1) [127]—are typically viewed as slowing the ageing rate. To reconcile these with the growth restriction model, we can consider the apparent paradox of body size and longevity. While larger species generally live longer than smaller species [39], larger individuals within a single species often have a shorter lifespan than smaller individuals [128,129]. In addition, larger individuals of a given species are typically at a higher risk for cancer [130,131]. It is worth noting that there are exceptions, such as interventions that lead to an increase in body size among individuals of the same species and to an increase in life expectancy, for example in marine bristle-worms [132].

The described lifespan-extending interventions could influence the mechanisms governing size variation *within* a species rather than the drivers of size differences *between* species. Pathways such as mTOR and GH signalling affect intra-species body size [133–136]. By contrast, larger, long-lived species often exhibit lower circulating IGF-1 levels [137] and evidence suggesting decreased mTOR signalling [138], which in turn suggests that they achieve larger sizes through independent mechanisms (figure 3). Consequently, growth restriction signalling as a driver of ageing may be more relevant to the inter-species relationship between body size, growth rate and longevity than the intra-specific differences in lifespan.

**Figure 3.**
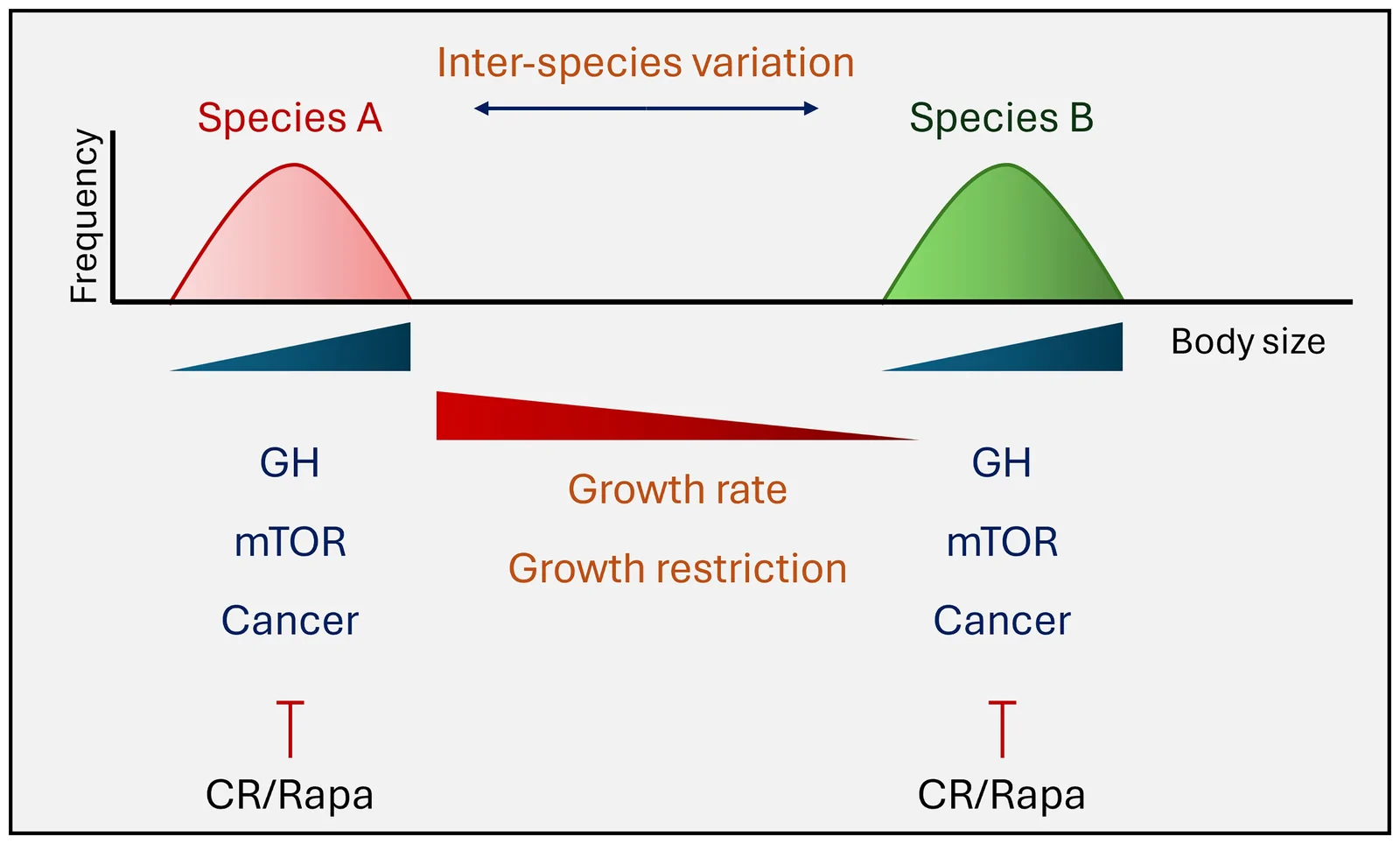
Representation of the proposed/conjectured relation between growth pathways, growth rate and body size. Schematic representation of the factors influencing growth rate, body size and lifespan in two hypothetical species (A and B). *x*-axes represent animal body size and *y*-axes represent the frequency in the population, representing a distribution of body sizes for species A and species B. Intraspecific variation: within a single species, larger body size is typically driven by processes including elevated GH signalling, increased mTOR activity. These pathways, while increasing size, are associated with a higher incidence of cancer and reduced lifespan. Such pathways can be modulated by interventions like CR or Rapa, which reduce growth and extend lifespan, potentially by mimicking the signalling profile of smaller individuals. Interspecific variation: by contrast, between species, larger body size (species B) is often achieved despite evidence of lower IGF1 signalling and reduced mTOR activity and is associated with a slower growth rate. This suggests that growth-restriction signalling may be less intense or delayed in larger species—our hypothesis—allowing for a more prolonged growth period before growth arrest and the emergence of ageing phenotypes. GH, growth hormone; mTOR, mammalian target of rapamycin; CR, caloric restriction; Rapa, rapamycin.

#### (ii) Developmental timing and signalling

Another possibility is that growth-reducing interventions slow the overall pace of development [5]. By decelerating development, the transition into the ‘growth-restriction’ phase is delayed, thereby postponing its associated physiological decline. In this view, growth restriction is an intrinsic process; while endocrine signalling can accelerate or retard its progress, it does not alter the underlying ‘in-built settings’ or the ultimate outcomes of the process. For instance, a genetic modification that accelerates growth and maturation in the turquoise killifish leads to a reduction in lifespan, supporting the suggestion that changes in developmental rate lead to changes in lifespan [139].

Furthermore, the nature of the growth restriction signals themselves may play a role. The negative feedback model suggests that tissue growth itself leads to the signals that suppress growth [13]. One possibility is that faster growth owing to stimulatory GHs, excess nutrients, or increased mTOR could trigger more intense growth-restriction signals, leading to greater tissue degradation during ageing. Therefore, the opposite is that the reduction of growth rate would lead to a less intense growth restriction later in life, with a lower level of deleterious effects.

#### (iii) The role of the mTOR in individuals of the same species

The interventions mentioned above (rapamycin, CR and mutations in GH/IGF1) act, at least in part, through the mTOR signalling axis [140–142]. It is possible that promoting growth is deleterious when achieved by systemically overactivating this specific pathway during development. Because this pathway is highly pleiotropic, its sustained activity may drive ageing phenotypes like cellular senescence [143] and age-associated diseases independently or in parallel to the effect of the pathway on growth itself.

#### (iv) Small long-lived species

Small animals that exceed their predicted lifespan based on body size, such as bats, present a challenge to the growth restriction model. Despite their small size, bats are remarkably long-lived, appearing as outliers to the standard correlation between body size and lifespan [39]. Another example is the axolotl, which is not only small and has a long lifespan for its size, but also possesses remarkable regenerative abilities. Furthermore, its epigenetic clock appears to stop ticking after development [144]. Another example of an organism with great developmental plasticity, for example, allowing for the re-initiation of growth as part of the regenerative process after injury (able to regenerate virtually an entire body), is the planarian [145]. These organisms may have evolved mechanisms to decouple growth regulation from the ageing process. Specifically, they might restrict growth while mitigating its proposed negative effects—perhaps by preventing growth rates from reaching detrimental ‘negative’ levels and instead stabilizing it at a rate close to optimal for tissue renewal.

### (d) Testing the growth restriction model and experimental challenges

The growth restriction model suggests that the processes responsible for decelerating tissue growth remain active into adulthood, eventually driving tissue atrophy. To test this model, preferably, would require manipulating these signals to maintain tissues at an ‘optimal’ homeostatic growth level, the renewal rate. If such stabilization could prevent atrophy and functional loss, it would provide evidence that persistent growth-restriction signalling is a contributing driver of ageing.

Currently, our ability to test this model is limited because the signals and molecular processes that mediate growth restriction remain largely unknown. Furthermore, these signalling systems are highly robust and likely redundant, having evolved to ensure proper body size. Understanding growth-regulatory processes facilitates the development of experimental designs needed to evaluate the growth-restriction hypothesis.

### (e) Possible experimental strategies

Despite the limitations, we can attempt experimental tests with current knowledge on tissue growth and cell proliferation regulation. If growth restriction causes some forms of age-related decline, then promoting controlled growth should prevent this loss of function. In proliferative tissues, this involves promoting cell division and molecular biosynthesis, while in quiescent tissues, this involves stimulating molecular biosynthesis (cell growth) without cell division. This could be tested by expressing cell proliferation regulators, such as the transcription factors (e.g. *Myc*, *E2F* or others), by activating local growth factors (e.g. fibroblast or endothelial growth factors) in old tissues or by manipulating developmental signalling pathways (such as the Wnt pathway), including telomerase activation. However, a potential undesirable side effect that must be taken into account is the increased risk of cancer. The interventions described above should reverse some age-associated phenotypes, such as tissue atrophy. In parallel, the opposite can be tested: suppression of proliferation-associated factors should accelerate ageing phenotypes, such as tissue atrophy.

The efficacy of growth manipulation would be evaluated through tissue-specific functional assays, alongside the assessment of ageing phenotypes, macromolecular biosynthesis measurements and potential reductions in ‘biological age’ as measured by epigenetic or transcriptomic clocks. The most accessible initial strategy involves *in vitro* experiments using cell cultures and tissue explants. Subsequent validation would require *in vivo* studies in animal models. At the organismal level, this approach necessitates either multi-tissue or systemic interventions capable of modulating growth rates independently of the growth hormone (GH/IGF1) pathway.

Alternatively, high-throughput screens could identify pharmacological agents that activate growth pathways, for example, using the restoration of youthful cell proliferation ability as a readout. Such drugs would provide a means to test whether stimulated tissue growth can reverse age-associated functional decline. Ideally, these pharmacological treatments would be adjustable, allowing for the promotion of tissue renewal while avoiding the induction of apoptosis or malignancy.

While our current understanding of growth regulation is incomplete, a fact that limits our experimental design capabilities, targeted manipulation of already known proliferation factors and local growth signalling provides preliminary experimental opportunities. By modulating these factors, we can generate empirical data to either support or challenge the growth restriction model. Such experiments, though limited, are essential for determining whether the suppression of growth serves as a fundamental driver of the ageing process or not.

## 6. Conclusions

The fundamental question of why we age has led to the development of numerous theories [1]. The coexistence of these competing models reflects our incomplete understanding of the ageing process; resolving this mystery is vital not only for biological theory but for global health. At present, we lack therapies that meaningfully modulate the underlying ageing process in humans.

In this work, we revisit the Bidder hypothesis, which proposes that growth restriction is a primary cause of ageing. We outline the processes by which growth restriction could lead to impaired tissue function and ageing phenotypes, evaluate current supporting evidence and judge this framework in relation to alternative theories. Furthermore, we address existing limitations and conflicting data. We expect this synthesis to highlight the merits of the model, as we consider that the collective evidence suggests that growth restriction is a plausible driver of the ageing process.

Overall, the hypothesis that ageing is a result of the continuation of growth restriction, first developed by Bidder, can be summarized as follows: one of the final stages of development is growth arrest, which results from a gradual decline in growth rate. This decline does not stop at the point of tissue renewal (where tissue production and degradation are balanced) but continues beyond it. When the growth rate becomes negative, it leads to tissue atrophy and functional decline. We suggest that this continued decline in growth rate may represent a contributing cause of ageing. We suggest that the growth restriction model is plausible and deserves further research.

## Data Availability

This article has no additional data.

## Theme

One contribution of 21 to a theme issue ‘Developmental circuits in ageing’.

## References

[1] de Magalhães JP . 2025 An overview of contemporary theories of ageing. Nat. Cell Biol. 27, 1074–1082. (doi:10.1038/s41556-025-01698-7) 40595368

[2] Gladyshev VN *et al.* 2024 Disagreement on foundational principles of biological aging. PNAS Nexus 3, 499. (doi:10.1093/pnasnexus/pgae499) PMC11630784 39660064

[3] Pamplona R , Jové M, Gómez J, Barja G. 2023 Programmed versus non-programmed evolution of aging. What is the evidence? Exp. Gerontol. 175, 112162. (doi:10.1016/j.exger.2023.112162) 37004927

[4] Goldsmith TC . 2008 Aging, evolvability, and the individual benefit requirement; medical implications of aging theory controversies. J. Theor. Biol. 252, 764–768. (doi:10.1016/j.jtbi.2008.02.035) 18396295

[5] de Magalhães JP . 2023 Ageing as a software design flaw. Genome Biol. 24, 24–51. (doi:10.1186/s13059-023-02888-y) PMC10042583 36973715

[6] Horvath S , Raj K. 2018 DNA methylation-based biomarkers and the epigenetic clock theory of ageing. Nat. Rev. Genet. 19, 371–384. (doi:10.1038/s41576-018-0004-3) 29643443

[7] Gems D , Virk RS, de Magalhães JP. 2024 Epigenetic clocks and programmatic aging. Ageing Res. Rev. 101, 102546. (doi:10.1016/j.arr.2024.102546) PMC7618570 39414120

[8] Bidder GP . 1932 Senescence. Br. Med. J. 2, 583–585. (doi:10.1136/bmj.2.3742.583) PMC2521615 20777068

[9] Martin GM . 1993 Clonal attenuation: causes and consequences. J. Gerontol. Biol. Sci. 48, B171–B172. (doi:10.1093/geronj/48.5.B171) 8366255

[10] Lui JC , Chen W, Barnes KM, Baron J. 2010 Changes in gene expression associated with aging commonly originate during juvenile growth. Mech. Ageing Dev. 131, 641–649. (doi:10.1016/j.mad.2010.08.010) PMC2956763 20816690

[11] Reddien PW . 2024 The purpose and ubiquity of turnover. Cell 187, 2657–2681. (doi:10.1016/j.cell.2024.04.034) 38788689

[12] Grantz KL *et al.* 2018 Fetal growth velocity: the NICHD fetal growth studies. Am. J. Obstet. Gynecol. 219, 285.e1–285.e36. (doi:10.1016/j.ajog.2018.05.016) PMC7035912 29803819

[13] Lui JC , Baron J. 2011 Mechanisms limiting body growth in mammals. Endocr. Rev. 32, 422–440. (doi:10.1210/er.2011-0001) PMC3365796 21441345

[14] Williams GC . 1957 Pleiotropy, natural selection, and the evolution of senescence. Evolution 11, 398–411. (doi:10.1111/j.1558-5646.1957.tb02911.x)

[15] Belikov AV . 2019 Age-related diseases as vicious cycles. Ageing Res. Rev. 49, 11–26. (doi:10.1016/j.arr.2018.11.002) 30458244

[16] Conlon I , Raff MC. 1999 Size control in animal development. Cell 96, 235–244. (doi:10.1016/S0092-8674(00)80563-2) 9988218

[17] Raff MC . 1996 Size control: the regulation of cell numbers in animal development. Cell 86, 173–175. (doi:10.1016/S0092-8674(00)80087-2) 8706120

[18] Ruijtenberg S , van den Heuvel S. 2016 Coordinating cell proliferation and differentiation: antagonism between cell cycle regulators and cell type-specific gene expression. Cell Cycle 15, 196–212. (doi:10.1080/15384101.2015.1120925) PMC4825819 26825227

[19] Soto AM , Longo G, Miquel PA, Montevil M, Mossio M, Perret N, Pocheville A, Sonnenschein C. 2016 Toward a theory of organisms: three founding principles in search of a useful integration. Prog. Biophys. Mol. Biol. 122, 77–82. (doi:10.1016/j.pbiomolbio.2016.07.006) PMC5097676 27498204

[20] Soto AM , Sonnenschein C. 2011 The tissue organization field theory of cancer: a testable replacement for the somatic mutation theory. Bioessays 33, 332–340. (doi:10.1002/bies.201100025) PMC3933226 21503935

[21] Hariharan IK , Wake DB, Wake MH. 2016 Indeterminate growth: could it represent the ancestral condition? Cold Spring Harb. Perspect. Biol. 8, a019174. (doi:10.1101/cshperspect.a019174) PMC4743077 26216720

[22] Vaupel JW , Baudisch A, Dölling M, Roach DA, Gampe J. 2004 The case for negative senescence. Theor. Popul. Biol. 65, 339–351. (doi:10.1016/j.tpb.2003.12.003) 15136009

[23] Guo P , Wan S, Guan Kl. 2025 The hippo pathway: organ size control and beyond. Pharmacol. Rev. 77, 100031. (doi:10.1016/j.pharmr.2024.100031) 40148032

[24] Nusse R , Clevers H. 2017 Wnt/β-catenin signaling, disease, and emerging therapeutic modalities. Cell 169, 985–999. (doi:10.1016/j.cell.2017.05.016) 28575679

[25] Massagué J , Sheppard D. 2023 TGF-β signaling in health and disease. Cell 186, 4007–4037. (doi:10.1016/j.cell.2023.07.036) PMC10772989 37714133

[26] Levin M , Stevenson CG. 2012 Regulation of cell behavior and tissue patterning by bioelectrical signals: challenges and opportunities for biomedical engineering. Annu. Rev. Biomed. Eng. 14, 295–323. (doi:10.1146/annurev-bioeng-071811-150114) PMC10472538 22809139

[27] Bonasio R , Tu S, Reinberg D. 2010 Molecular signals of epigenetic states. Science 330, 612–616. (doi:10.1126/science.1191078) PMC3772643 21030644

[28] Duronio RJ , Xiong Y. 2013 Signaling pathways that control cell proliferation. Cold Spring Harb. Perspect. Biol. 5, a008904. (doi:10.1101/cshperspect.a008904) PMC3578363 23457258

[29] Lam I , Pickering CM, Mac Gabhann F. 2019 Context-dependent regulation of receptor tyrosine kinases: insights from systems biology approaches. Wiley Interdiscip. Rev. Syst. Biol. Med. 11, e1437. (doi:10.1002/wsbm.1437) PMC6537588 30255986

[30] Gokhale RH , Shingleton AW. 2015 Size control: the developmental physiology of body and organ size regulation. Wiley Interdiscip. Rev. Dev. Biol. 4, 335–356. (doi:10.1002/wdev.181) 25808999

[31] Belugali Nataraj N , Yarden Y. 2021Growth factors. In Encyclopedia of molecular pharmacology (eds Offermanns S, Rosenthal W), pp. 1–13. Cham, Switzerland: Springer International Publishing. (doi:10.1007/978-3-030-21573-6_160-1)

[32] Huang X , Sun D, Wu T, Liu X, Xu S, Yang G. 2021 Genomic insights into body size evolution in Carnivora support Peto's paradox. BMC Genom. 22, 429. (doi:10.1186/s12864-021-07732-w) PMC8191207 34107880

[33] Tollis M *et al.* 2019 Return to the sea, get huge, beat cancer: an analysis of cetacean genomes including an assembly for the humpback whale (*Megaptera novaeangliae*). Mol. Biol. Evol. 36, 1746–1763. (doi:10.1093/molbev/msz099) PMC6657726 31070747

[34] Tejada-Martinez D , de Magalhães JP, Opazo JC. 2021 Positive selection and gene duplications in tumour suppressor genes reveal clues about how cetaceans resist cancer. Proc. R. Soc. B 288, 20202592. (doi:10.1098/rspb.2020.2592) PMC7935004 33622125

[35] Sun D , Chai S, Huang X, Wang Y, Xiao L, Xu S, Yang G. 2022 Novel genomic insights into body size evolution in cetaceans and a resolution of Peto's paradox. Am. Nat. 199, E28–E42. (doi:10.1086/717768) 35077272

[36] Sun Y , Liu Y, Sun X, Lin Y, Yin D, Xu S, Yang G. 2019 Insights into body size variation in cetaceans from the evolution of body-size-related genes. BMC Evol. Biol. 19, 157. (doi:10.1186/s12862-019-1461-9) PMC6660953 31351448

[37] Xia T *et al.* 2025 Genomic insights into the mechanisms of body size evolution in Serpentes. BMC Genom. 26, 420. (doi:10.1186/s12864-025-11601-1) PMC12039104 40301758

[38] Herrera-Álvarez S , Karlsson E, Ryder OA, Lindblad-Toh K, Crawford AJ. 2021 How to make a rodent giant: genomic basis and tradeoffs of gigantism in the capybara, the world's largest rodent. Mol. Biol. Evol. 38, 1715–1730. (doi:10.1093/molbev/msaa285) PMC8097284 33169792

[39] de Magalhães JP , Costa J, Church GM. 2007 An analysis of the relationship between metabolism, developmental schedules, and longevity using phylogenetic independent contrasts. J. Gerontol. A 62, 149–160. (doi:10.1093/gerona/62.2.149) PMC2288695 17339640

[40] Binter J , Nobis MP, Altman J, Büntgen U, Crivellaro A, Liancourt P, Doležal J. 2026 Slow growth as a strategy for plant longevity. Nat. Commun. 17, 9703. (doi:10.1038/s41467-026-76765-0) PMC13562615 42722672

[41] Liang Z , Dong X, Zhang Z, Zhang Q, Zhao Y. 2022 Age-related thymic involution: mechanisms and functional impact. Aging Cell 21, e13671. (doi:10.1111/acel.13671) PMC9381902 35822239

[42] Bethlehem RAI *et al.* 2022 Brain charts for the human lifespan. Nature 604, 525–533. (doi:10.1038/s41586-022-04554-y) PMC9021021 35388223

[43] Habes M *et al.* 2016 Advanced brain aging: relationship with epidemiologic and genetic risk factors, and overlap with Alzheimer disease atrophy patterns. Transl. Psychiatry 6, e775. (doi:10.1038/tp.2016.39) PMC4872397 27045845

[44] Borzage M , Blüml S, Seri I. 2014 Equations to describe brain size across the continuum of human lifespan. Brain Struct. Funct. 219, 141–150. (doi:10.1007/s00429-012-0490-6) 23239013

[45] Blinkouskaya Y , Caçoilo A, Gollamudi T, Jalalian S, Weickenmeier J. 2021 Brain aging mechanisms with mechanical manifestations. Mech. Ageing Dev. 200, 111575. (doi:10.1016/j.mad.2021.111575) PMC8627478 34600936

[46] Walløe S , Pakkenberg B, Fabricius K. 2014 Stereological estimation of total cell numbers in the human cerebral and cerebellar cortex. Front. Hum. Neurosci. 8, 508. (doi:10.3389/fnhum.2014.00508) PMC4097828 25076882

[47] Shaw SC , Dennison EM, Cooper C. 2017 Epidemiology of sarcopenia: determinants throughout the lifecourse. Calcif. Tissue Int. 101, 229–247. (doi:10.1007/s00223-017-0277-0) PMC5544114 28421264

[48] Fielding RA *et al.* 2011 Sarcopenia: an undiagnosed condition in older adults. Current consensus definition: prevalence, etiology, and consequences. International working group on sarcopenia. J. Am. Med. Dir. Assoc. 12, 249–256. (doi:10.1016/j.jamda.2011.01.003) PMC3377163 21527165

[49] Nilwik R , Snijders T, Leenders M, Groen BBL, van Kranenburg J, Verdijk LB, van Loon LJC. 2013 The decline in skeletal muscle mass with aging is mainly attributed to a reduction in type II muscle fiber size. Exp. Gerontol. 48, 492–498. (doi:10.1016/j.exger.2013.02.012) 23425621

[50] Wilkinson DJ , Piasecki M, Atherton PJ. 2018 The age-related loss of skeletal muscle mass and function: measurement and physiology of muscle fibre atrophy and muscle fibre loss in humans. Ageing Res. Rev. 47, 123–132. (doi:10.1016/j.arr.2018.07.005) PMC6202460 30048806

[51] Griffith JF . 2017 Age-related changes in the bone marrow. Curr. Radiol. Rep. 5, 24. (doi:10.1007/s40134-017-0218-8)

[52] Sfeir JG , Drake MT, Khosla S, Farr JN. 2022 Skeletal aging. Mayo Clin. Proc. 97, 1194–1208. (doi:10.1016/j.mayocp.2022.03.011) PMC9179169 35662432

[53] Roseman DA , Hwang S-J, Oyama-Manabe N, Chuang ML, O'Donnell CJ, Manning WJ, Fox CS. 2017 Clinical associations of total kidney volume: the Framingham heart study. Nephrol. Dial. Transplant. 32, 1344–1350. (doi:10.1093/ndt/gfw237) PMC5837692 27325252

[54] Wang X , Vrtiska TJ, Avula RT, Walters LR, Chakkera HA, Kremers WK, Lerman LO, Rule AD. 2014 Age, kidney function, and risk factors associate differently with cortical and medullary volumes of the kidney. Kidney Int. 85, 677–685. (doi:10.1038/ki.2013.359) PMC3943620 24067437

[55] Denic A , Lieske JC, Chakkera HA, Poggio ED, Alexander MP, Singh P, Kremers WK, Lerman LO, Rule AD. 2017 The substantial loss of nephrons in healthy human kidneys with aging. J. Am. Soc. Nephrol. 28, 313–320. (doi:10.1681/ASN.2016020154) PMC5198286 27401688

[56] Gourtsoyiannis N , Prassopoulos P, Cavouras D, Pantelidis N. 1990 The thickness of the renal parenchyma decreases with age: a CT study of 360 patients. Am. J. Roentgenol. 155, 541–544. (doi:10.2214/ajr.155.3.2117353) 2117353

[57] Branchet M , Boisnic S, Frances C, Robert A. 1990 Skin thickness changes in normal aging skin. Gerontology 36, 28–35. (doi:10.1159/000213172) 2384222

[58] Zou Z *et al.* 2021 A single-cell transcriptomic atlas of human skin aging. Dev. Cell 56, 383–397. (doi:10.1016/j.devcel.2020.11.002) 33238152

[59] Han KS *et al.* 2024 qMAP enabled microanatomical mapping of human skin aging. bioRxiv. (doi:10.1101/2024.04.03.588011)

[60] Saisho Y , Butler AE, Meier JJ, Monchamp T, Allen‐Auerbach M, Rizza RA, Butler PC. 2007 Pancreas volumes in humans from birth to age one hundred taking into account sex, obesity, and presence of type-2 diabetes. Clin. Anat. 20, 933–942. (doi:10.1002/ca.20543) PMC2680737 17879305

[61] Gregg BE , Moore PC, Demozay D, Hall BA, Li M, Husain A, Wright AJ, Atkinson MA, Rhodes CJ. 2012 Formation of a human β-cell population within pancreatic islets is set early in life. J. Clin. Endocrinol. Metab. 97, 3197–3206. (doi:10.1210/jc.2012-1206) PMC3431572 22745242

[62] Gomez-Pinilla PJ *et al.* 2011 Changes in interstitial cells of cajal with age in the human stomach and colon. Neurogastroenterol. Motil. 23, 36–44. (doi:10.1111/j.1365-2982.2010.01590.x) PMC2999641 20723073

[63] Baidoo N , Sanger GJ. 2024 Age-related decline in goblet cell numbers and mucin content of the human colon: implications for lower bowel functions in the elderly. Exp. Mol. Pathol. 139, 104923. (doi:10.1016/j.yexmp.2024.104923) 39154390

[64] Vara-Luiz F *et al.* 2026 Age-related decline in intestinal villus length: a cross-sectional study on the human gut. Nutrients 18, 1172. (doi:10.3390/nu18081172) PMC13118423 42074985

[65] Wynne HA , Cope LH, Mutch E, Rawlins MD, Woodhouse KW, James OFW. 1989 The effect of age upon liver volume and apparent liver blood flow in healthy man. Hepatology 9, 297–301. (doi:10.1002/hep.1840090222) 2643548

[66] Michalopoulos GK , Bhushan B. 2021 Liver regeneration: biological and pathological mechanisms and implications. Nat. Rev. Gastroenterol. Hepatol. 18, 40–55. (doi:10.1038/s41575-020-0342-4) 32764740

[67] Chatterjee N , Sharma R, Kale PR, Trehanpati N, Ramakrishna G. 2025 Is the liver resilient to the process of ageing? Ann. Hepatol. 30, 101580. (doi:10.1016/j.aohep.2024.101580) 39276981

[68] Heinke P *et al.* 2022 Diploid hepatocytes drive physiological liver renewal in adult humans. Cell Syst. 13, 499–507. (doi:10.1016/j.cels.2022.05.001) 35649419

[69] Si L *et al.* 2021 Knee cartilage thickness differs alongside ages: a 3-T magnetic resonance research upon 2,481 subjects via deep learning. Front. Med. 7, 600049. (doi:10.3389/fmed.2020.600049) PMC7900571 33634142

[70] Cai G , Jiang M, Cicuttini F, Jones G. 2019 Association of age, sex and BMI with the rate of change in tibial cartilage volume: a 10.7-year longitudinal cohort study. Arthritis Res. Ther. 21, 273. (doi:10.1186/s13075-019-2063-z) PMC6902563 31818318

[71] Machino M , Nakashima H, Ito K, Tsushima M, Ando K, Kobayashi K, Imagama S. 2021 Influence of age and gender on intervertebral disk degeneration and height in the thoracolumbar spine. Spine Surg. Relat. Res. 6, 379–387. (doi:10.22603/ssrr.2021-0187) PMC9381080 36051683

[72] Videman T , Battié MC, Gibbons LE, Gill K. 2014 Aging changes in lumbar discs and vertebrae and their interaction: a 15-year follow-up study. Spine J. 14, 469–478. (doi:10.1016/j.spinee.2013.11.018) 24262855

[73] Yoneshiro T , Aita S, Matsushita M, Okamatsu‐Ogura Y, Kameya T, Kawai Y, Miyagawa M, Tsujisaki M, Saito M. 2011 Age-related decrease in cold-activated brown adipose tissue and accumulation of body fat in healthy humans. Obesity 19, 1755–1760. (doi:10.1038/oby.2011.125) 21566561

[74] Caso G , McNurlan MA, Mileva I, Zemlyak A, Mynarcik DC, Gelato MC. 2013 Peripheral fat loss and decline in adipogenesis in older humans. Metabolism 62, 337–340. (doi:10.1016/j.metabol.2012.08.007) PMC3531563 22999012

[75] Enzi G , Gasparo M, Biondetti P, Fiore D, Semisa M, Zurlo F. 1986 Subcutaneous and visceral fat distribution according to sex, age, and overweight, evaluated by computed tomography. Am. J. Clin. Nutr. 44, 739–746. (doi:10.1093/ajcn/44.6.739) 3788827

[76] Pfannenberg C *et al.* 2010 Impact of age on the relationships of brown adipose tissue with sex and adiposity in humans. Diabetes 59, 1789–1793. (doi:10.2337/db10-0004) PMC2889780 20357363

[77] Wallace WHB , Kelsey TW. 2010 Human ovarian reserve from conception to the menopause. PLoS ONE 5, e8772. (doi:10.1371/journal.pone.0008772) PMC2811725 20111701

[78] Kelsey TW , Dodwell SK, Wilkinson AG, Greve T, Andersen CY, Anderson RA, Wallace WHB. 2013 Ovarian volume throughout life: a validated normative model. PLoS ONE 8, e71465. (doi:10.1371/journal.pone.0071465) PMC3760857 24019863

[79] Yang H *et al.* 2011 The effects of aging on testicular volume and glucose metabolism: an investigation with ultrasonography and FDG-PET. Mol. Imaging Biol. 13, 391–398. (doi:10.1007/s11307-010-0341-x) 20521135

[80] Ruggiero C *et al.* 2007 White blood cell count and mortality in the Baltimore Longitudinal Study of Aging. J. Am. Coll. Cardiol. 49, 1841–1850. (doi:10.1016/j.jacc.2007.01.076) PMC2646088 17481443

[81] Wang Z , Li L, Glicksberg BS, Israel A, Dudley JT, Ma'ayan A. 2017 Predicting age by mining electronic medical records with deep learning characterizes differences between chronological and physiological age. J. Biomed. Inform. 76, 59–68. (doi:10.1016/j.jbi.2017.11.003) PMC5716867 29113935

[82] Keng BMH , Gao F, Teo LLY, Lim WS, Tan RS, Ruan W, Ewe SH, Koh WP, Koh AS. 2019 Associations between skeletal muscle and myocardium in aging: a syndrome of ‘cardio-sarcopenia’? J. Am. Geriatr. Soc. 67, 2568–2573. (doi:10.1111/jgs.16132) PMC6898740 31418823

[83] Loh DR , Tan RS, Lim WS, Koh AS. 2022 Cardio-sarcopenia: a syndrome of concern in aging. Front. Med. 9, 1027466. (doi:10.3389/fmed.2022.1027466) PMC9640679 36388892

[84] Shiraishi I , Takamatsu T, Minamikawa T, Onouchi Z, Fujita S. 1992 Quantitative histological analysis of the human sinoatrial node during growth and aging. Circulation 85, 2176–2184. (doi:10.1161/01.CIR.85.6.2176) 1591834

[85] Thery C , Gosselin B, Lekieffre J, Warembourg H. 1977 Pathology of sinoatrial node. Correlations with electrocardiographic findings in 111 patients. Am. Heart J. 93, 735–740. (doi:10.1016/s0002-8703(77)80070-7) 871100

[86] Olivetti G , Giordano G, Corradi D, Melissari M, Lagrasta C, Gambert SR, Anversa P. 1995 Gender differences and aging: effects on the human heart. J. Am. Coll. Cardiol. 26, 1068–1079. (doi:10.1016/0735-1097(95)00282-8) 7560601

[87] Strait JB , Lakatta EG. 2012 Aging-associated cardiovascular changes and their relationship to heart failure. Heart Fail. Clin. 8, 143–164. (doi:10.1016/j.hfc.2011.08.011) PMC3223374 22108734

[88] Hees PS , Fleg JL, Lakatta EG, Shapiro EP. 2002 Left ventricular remodeling with age in normal men versus women: novel insights using three-dimensional magnetic resonance imaging. Am. J. Cardiol. 90, 1231–1236. (doi:10.1016/s0002-9149(02)02840-0) 12450604

[89] Lakatta EG , Levy D. 2003 Arterial and cardiac aging: major shareholders in cardiovascular disease enterprises: part II: the aging heart in health: links to heart disease. Circulation 107, 346–354. (doi:10.1161/01.CIR.0000048893.62841.F7) 12538439

[90] Verleden SE *et al.* 2021 Small airway loss in the physiologically ageing lung: a cross-sectional study in unused donor lungs. Lancet Respir. Med. 9, 167–174. (doi:10.1016/S2213-2600(20)30324-6) 33031747

[91] De Man R *et al.* 2023 A multi-omic analysis of the human lung reveals distinct cell specific aging and senescence molecular programs. bioRxiv. (doi:10.1101/2023.04.19.536722)

[92] Vilasboas-Campos V , Jammal AA, Gracitelli CPB, Gameiro GR, Costa VP, Medeiros FA. 2025 Estimated rates of retinal ganglion cell loss with aging. Ophthalmol. Sci. 5, 100616. (doi:10.1016/j.xops.2024.100616) PMC11584911 39584181

[93] Cavallotti C , Artico M, Pescosolido N, Leali FMT, Feher J. 2004 Age-related changes in the human retina. Can. J. Ophthalmol. 39, 61–68. (doi:10.1016/S0008-4182(04)80054-1) 15040616

[94] Galgauskas S , Norvydaite D, Krasauskaite D, Stech S, Ašoklis RS. 2013 Age-related changes in corneal thickness and endothelial characteristics. Clin. Interv. Aging. 8, 1445–1450. (doi:10.2147/CIA.S51693) PMC3810328 24187493

[95] Panda-Jonas S , Jonas JB, Jakobczyk-Zmija M. 1995 Retinal photoreceptor density decreases with age. Ophthalmology 102, 1853–1859. (doi:10.1016/S0161-6420(95)30784-1) 9098287

[96] Wu PZ , Liberman LD, Bennett K, de Gruttola V, O'Malley JT, Liberman MC. 2019 Primary neural degeneration in the human cochlea: evidence for hidden hearing loss in the aging ear. Neuroscience 407, 8–20. (doi:10.1016/j.neuroscience.2018.07.053) PMC6369025 30099118

[97] Vesely S , Knutson T, Damber JE, Dicuio M, Dahlstrand C. 2003 Relationship between age, prostate volume, prostate specific antigen, symptom score and uroflowmetry in men with lower urinary tract symptoms: does prostate size matter?. Scand. J. Urol. Nephrol. 37, 322–328. (doi:10.1080/00365590310014760) 12944191

[98] Feneley MR , Landis P, Simon I, Metter EJ, Morrell CH, Carter HB, Walsh PC. 2000 Today men with prostate cancer have larger prostates. Urology 56, 839–842. (doi:10.1016/s0090-4295(00)00738-x) 11068313

[99] Frenzel A , Binder H, Walter N, Wirkner K, Loeffler M, Loeffler-Wirth H. 2020 The aging human body shape. NPJ Aging Mech. Dis. 6, 5. (doi:10.1038/s41514-020-0043-9) PMC7093543 32218988

[100] Abila E *et al.* 2024 Histological aging signatures enable tissue-specific disease prediction from blood. bioRxiv. (doi:10.1101/2024.11.14.618081)

[101] Richardson RB , Allan DS, Le Y. 2014 Greater organ involution in highly proliferative tissues associated with the early onset and acceleration of ageing in humans. Exp. Gerontol. 55, 80–91. (doi:10.1016/j.exger.2014.03.015) 24685641

[102] Martin GM . 2007 The genetics and epigenetics of altered proliferative homeostasis in ageing and cancer. Mech. Ageing Dev. 128, 9–12. (doi:10.1016/j.mad.2006.11.003) PMC1868440 17116316

[103] Miller I , Min M, Yang C, Tian C, Gookin S, Carter D, Spencer SL. 2018 Ki67 is a graded rather than a binary marker of proliferation versus quiescence. Cell Rep. 24, 1105–1112. (doi:10.1016/j.celrep.2018.06.110) PMC6108547 30067968

[104] Tomasetti C *et al.* 2019 Cell division rates decrease with age, providing a potential explanation for the age-dependent deceleration in cancer incidence. Proc. Natl Acad. Sci. USA 116, 20 482–20 488. (doi:10.1073/pnas.1905722116) PMC6789572 31548407

[105] Vernon RY , William PS, Paul BP, Joerg CW, Nevin SS. 1975 Total human body protein synthesis in relation to protein requirements at various ages. Nature 253, 192–194. (doi:10.1038/253192a0) 1110767

[106] Thomas S . 1996 A longitudinal study of human age-related ribosomal RNA gene activity as detected by silver-stained NORs. Mech. Ageing Dev. 92, 101–109. (doi:10.1016/S0047-6374(96)01805-2) 9080391

[107] Rooyackers OE , Adey DB, Ades PA, Nair KS. 1996 Effect of age on *in vivo* rates of mitochondrial protein synthesis in human skeletal muscle. Proc. Natl Acad. Sci. USA 93, 15 364–15 369. (doi:10.1073/pnas.93.26.15364) PMC26410 8986817

[108] de Magalhães JP , Curado J, Church GM. 2009 Meta-analysis of age-related gene expression profiles identifies common signatures of aging. Bioinformatics 25, 875–881. (doi:10.1093/bioinformatics/btp073) PMC2732303 19189975

[109] Frenk S , Houseley J. 2018 Gene expression hallmarks of cellular ageing. Biogerontology 19, 547–566. (doi:10.1007/s10522-018-9750-z) PMC6223719 29492790

[110] Youm YH , Horvath TL, Mangelsdorf DJ, Kliewer SA, Dixit VD. 2016 Prolongevity hormone FGF21 protects against immune senescence by delaying age-related thymic involution. Proc. Natl Acad. Sci. USA 113, 1026–1031. (doi:10.1073/pnas.1514511113) PMC4743827 26755598

[111] Youm YH , Gliniak C, Zhang Y, Dlugos T, Scherer PE, Dixit VD. 2025 Enhanced paracrine action of FGF21 in stromal cells delays thymic aging. Nat. Aging 5, 576–587. (doi:10.1038/s43587-025-00813-5) PMC12003152 39972172

[112] Grunewald M *et al.* 2021 Counteracting age-related VEGF signaling insufficiency promotes healthy aging and extends life span. Science 373, E3692–E3701. (doi:10.1126/science.abc8479) 34326210

[113] Keren A , Bertolini M, Keren Y, Ullmann Y, Paus R, Gilhar A. 2022 Human organ rejuvenation by VEGF-A: lessons from the skin. Sci. Adv. 8, 6756. (doi:10.1126/sciadv.abm6756) PMC9232104 35749494

[114] Endo Y , Hwang CD, Zhang Y, Olumi S, Koh DJ, Zhu C, Neppl RL, Agarwal S, Sinha I. 2023 VEGFA promotes skeletal muscle regeneration in aging. Adv. Biol. 7, e2200320. (doi:10.1002/adbi.202200320) PMC10539483 36988414

[115] Jha RK , Kouzine F, Levens D. 2023 MYC function and regulation in physiological perspective. Front. Cell Dev. Biol. 11, 1268275. (doi:10.3389/fcell.2023.1268275) PMC10627926 37941901

[116] Wang H *et al.* 2023 Premature aging and reduced cancer incidence associated with near-complete body-wide Myc inactivation. Cell Rep. 42, 112830. (doi:10.1016/j.celrep.2023.112830) PMC10591215 37481724

[117] Macedo JC *et al.* 2018 FoxM1 repression during human aging leads to mitotic decline and aneuploidy-driven full senescence. Nat. Commun. 9, 2834. (doi:10.1038/s41467-018-05258-6) PMC6053425 30026603

[118] Ribeiro R *et al.* 2022 *In vivo* cyclic induction of the FOXM1 transcription factor delays natural and progeroid aging phenotypes and extends healthspan. Nat. Aging 2, 397–411. (doi:10.1038/s43587-022-00209-9) 37118067

[119] Wolf AM . 2021 The tumor suppression theory of aging. Mech. Ageing Dev. 200, 111583. (doi:10.1016/j.mad.2021.111583) 34637937

[120] de Magalhães JP . 2025 The evolution of cancer and ageing: a history of constraint. Nat. Rev. Cancer 25, 873–880. (doi:10.1038/s41568-025-00861-4) 40859047

[121] Wordsworth J , de Nielsen PY, Fielder E, Chandrasegaran S, Shanley D. 2023 Metabolic slowdown as the proximal cause of ageing and death. bioRxiv. (doi:10.1101/2023.08.01.551537)

[122] Wordsworth J , O’ Keefe H, Clark P, Shanley D. 2022 The damage-independent evolution of ageing by selective destruction. Mech. Ageing Dev. 111709, 207. (doi:10.1016/j.mad.2022.111709) 35868541

[123] Blagosklonny MV . 2006 Aging and immortality: quasi-programmed senescence and its pharmacologic inhibition. Cell Cycle 5, 2087–2102. (doi:10.4161/cc.5.18.3288) 17012837

[124] Blagosklonny MV . 2009 TOR-driven aging: speeding car without brakes. Cell Cycle 8, 4055–4059. (doi:10.4161/cc.8.24.10310) 19923900

[125] Di Francesco A *et al.* 2024 Dietary restriction impacts health and lifespan of genetically diverse mice. Nature 634, 684–692. (doi:10.1038/s41586-024-08026-3) PMC11485257 39385029

[126] Ivimey‐Cook ER , Sultanova Z, Maklakov AA. 2025 Rapamycin, not metformin, mirrors dietary restriction-driven lifespan extension in vertebrates: a meta-analysis. Aging Cell 24, e70131. (doi:10.1111/acel.70131) PMC12419861 40532901

[127] Kenyon CJ . 2010 The genetics of ageing. Nature 464, 504–512. (doi:10.1038/nature08980) 20336132

[128] Miller RA , Harper JM, Galecki A, Burke DT. 2002 Big mice die young: early life body weight predicts longevity in genetically heterogeneous mice. Aging Cell 1, 22–29. (doi:10.1046/j.1474-9728.2002.00006.x) 12882350

[129] Kraus C , Pavard SL, Promislow DE. 2013 The size-life span trade-off decomposed: why large dogs die young. Am. Nat. 181, 492–505. (doi:10.1086/669665) 23535614

[130] Doherty A , Lopes I, Ford CT, Monaco G, Guest P, de Magalhães JP. 2020 A scan for genes associated with cancer mortality and longevity in pedigree dog breeds. Mamm. Genome 31, 215–227. (doi:10.1007/s00335-020-09845-1) PMC7496057 32661568

[131] Nunney L . 2018 Size matters: height, cell number and a person's risk of cancer. Proc. R. Soc. B 285, 20181743. (doi:10.1098/rspb.2018.1743) PMC6234890 30355711

[132] Andreatta G , Scaramuzza F, Ćorić A, Orel L, Mat AM, Takekata H, Poehn B, Milivojev N, Tessmar-Raible K. 2026 Photoreceptor control of *Platynereis* growth and lifespan via evolutionarily conserved molecular pathways. Proc. Natl Acad. Sci. USA 123, e2514719123. (doi:10.1073/pnas.2514719123) PMC13012086 41855267

[133] Zhou Y *et al.* 1997 A mammalian model for Laron syndrome produced by targeted disruption of the mouse growth hormone receptorbinding protein gene (the Laron mouse). Proc. Natl Acad. Sci. USA 94, 13 215–13 220. (doi:10.1073/pnas.94.24.13215) PMC24289 9371826

[134] Rimbault M , Beale HC, Schoenebeck JJ, Hoopes BC, Allen JJ, Kilroy-Glynn P, Wayne RK, Sutter NB, Ostrander EA. 2013 Derived variants at six genes explain nearly half of size reduction in dog breeds. Genome Res. 23, 1985–1995. (doi:10.1101/gr.157339.113) PMC3847769 24026177

[135] Leontieva OV , Paszkiewicz G, Blagosklonny MV. 2012 Mechanistic or mammalian target of rapamycin (mTOR) may determine robustness in young male mice at the cost of accelerated aging. Aging 4, 899–916. (doi:10.18632/aging.100528) PMC3615157 23443503

[136] Shindyapina AV *et al.* 2022 Rapamycin treatment during development extends lifespan and healthspan. bioRxiv. (doi:10.1101/2022.02.18.481092)

[137] Stuart JA , Page MM. 2010 Plasma IGF-1 is negatively correlated with body mass in a comparison of 36 mammalian species. Mech. Ageing Dev. 131, 591–598. (doi:10.1016/j.mad.2010.08.005) 20736031

[138] Jové M , Mota-Martorell N, Fernàndez-Bernal A, Portero-Otin M, Barja G, Pamplona R. 2023 Phenotypic molecular features of long-lived animal species. Free Radic. Biol. Med. 208, 728–747. (doi:10.1016/j.freeradbiomed.2023.09.023) 37748717

[139] Moses E *et al.* 2026 An antagonistically pleiotropic gene regulates vertebrate growth, maturity, and lifespan. Nat. Commun. 17, 4410. (doi:10.1038/s41467-026-72381-0) PMC13230779 42230577

[140] Liu GY , Sabatini DM. 2020 mTOR at the nexus of nutrition, growth, ageing and disease. Nat. Rev. Mol. Cell Biol. 21, 183–203. (doi:10.1038/s41580-019-0199-y) PMC7102936 31937935

[141] Laplante M , Sabatini DM. 2012 mTOR signaling in growth control and disease. Cell 149, 274–293. (doi:10.1016/j.cell.2012.03.017) PMC3331679 22500797

[142] Kim J , Guan KL. 2019 mTOR as a central hub of nutrient signalling and cell growth. Nat. Cell Biol. 21, 63–71. (doi:10.1038/s41556-018-0205-1) 30602761

[143] Blagosklonny MV . 2018 Rapamycin, proliferation and geroconversion to senescence. Cell Cycle 17, 2655–2665. (doi:10.1080/15384101.2018.1554781) PMC6343718 30541374

[144] Haluza Y *et al.* 2024 Axolotl epigenetic clocks offer insights into the nature of negligible senescence. bioRxiv. (doi:10.1101/2024.09.09.611397)

[145] Reddien PW . 2018 The cellular and molecular basis for planarian regeneration. Cell 175, 327–345. (doi:10.1016/j.cell.2018.09.021) PMC7706840 30290140

